# A 28-Year-Old Woman With Chest Pain, Abdominal Pain, and Right Pleural Effusion

**DOI:** 10.1016/j.chpulm.2024.100064

**Published:** 2024-05-23

**Authors:** Rohit Shirgaonkar, Gouda Raja, Mohammed Shahin, Sweta Singh, Amit Kumar Adhya, Manoj Kumar Panigrahi

**Affiliations:** a Departments of Pulmonary Medicine & Critical Care, All India Institute of Medical Sciences, Bhubaneswar, India; b Obstetrics and Gynecology, All India Institute of Medical Sciences, Bhubaneswar, India; c Pathology and Laboratory Medicine, All India Institute of Medical Sciences, Bhubaneswar, India

## Abstract

A 28-year-old woman presented to the outpatient setting with right-sided intermittent chest pain for the past 8 months. For the past 3 months, she noticed breathlessness initially on exertion, which had progressed to dyspnea at rest. She also reported intermittent cramping abdominal pain, predominantly in the pelvis, that worsened during each menstrual cycle. She had regular menses and denied the use of any hormonal-based or barrier methods of contraception. She had never smoked. She was married and delivered a child 10 years ago. Two years before this presentation, she had undergone medical termination of pregnancy. A previous Pap smear of the cervix was normal. She denied any history of cough, hemoptysis, vomiting, diarrhea or hematemesis, dyspareunia, or post-coital bleeding. There was no relevant family history.

## Physical Examination Findings

The patient was conscious and well oriented at presentation and had mild respiratory distress from using accessory muscles. Her vitals were as follows: heart rate of 104 beats/min, BP of 106/80 mm Hg, respiratory rate of 30 breaths/min, and oxygen saturation of 97% on ambient air. A physical examination of the chest showed reduced tactile fremitus, dull percussion notes, and reduced intensity breath sounds on the right side. Palpation of the abdomen revealed minimal tenderness in the suprapubic area. Examination of the cardiovascular and central nervous systems was normal. Pelvic examination showed the uterus was anteverted with restricted mobility. The bilateral adnexa were free, and a nodular, nontender lesion approximately 2 × 2 cm was palpable posteriorly in the pouch of Douglas.

## Diagnostic Studies

**Figure 1:**
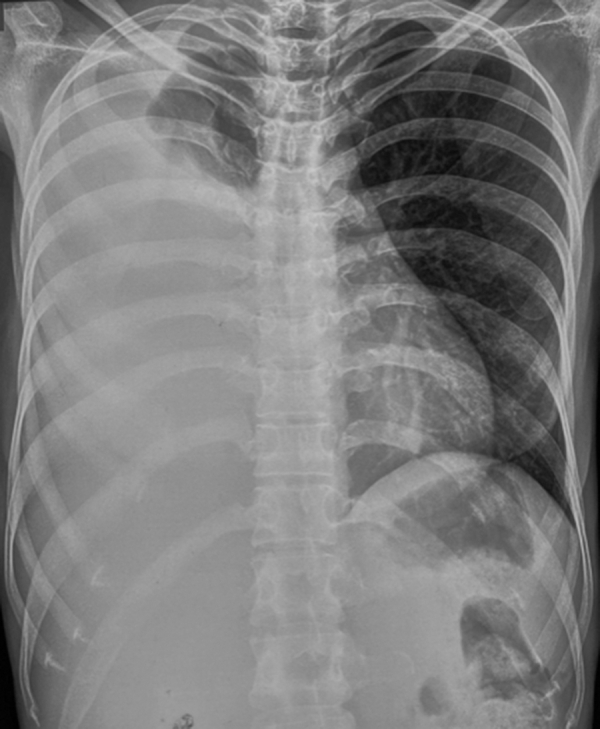
Chest radiograph posteroanterior view shows an opaque right hemithorax with a contralateral shift of the mediastinum.

**Figure 2:**
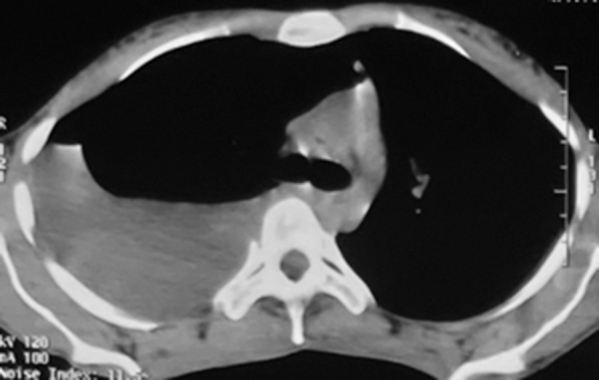
CT scan of the chest shows a hypodense opacity on the right hemithorax.

**Figure 3:**
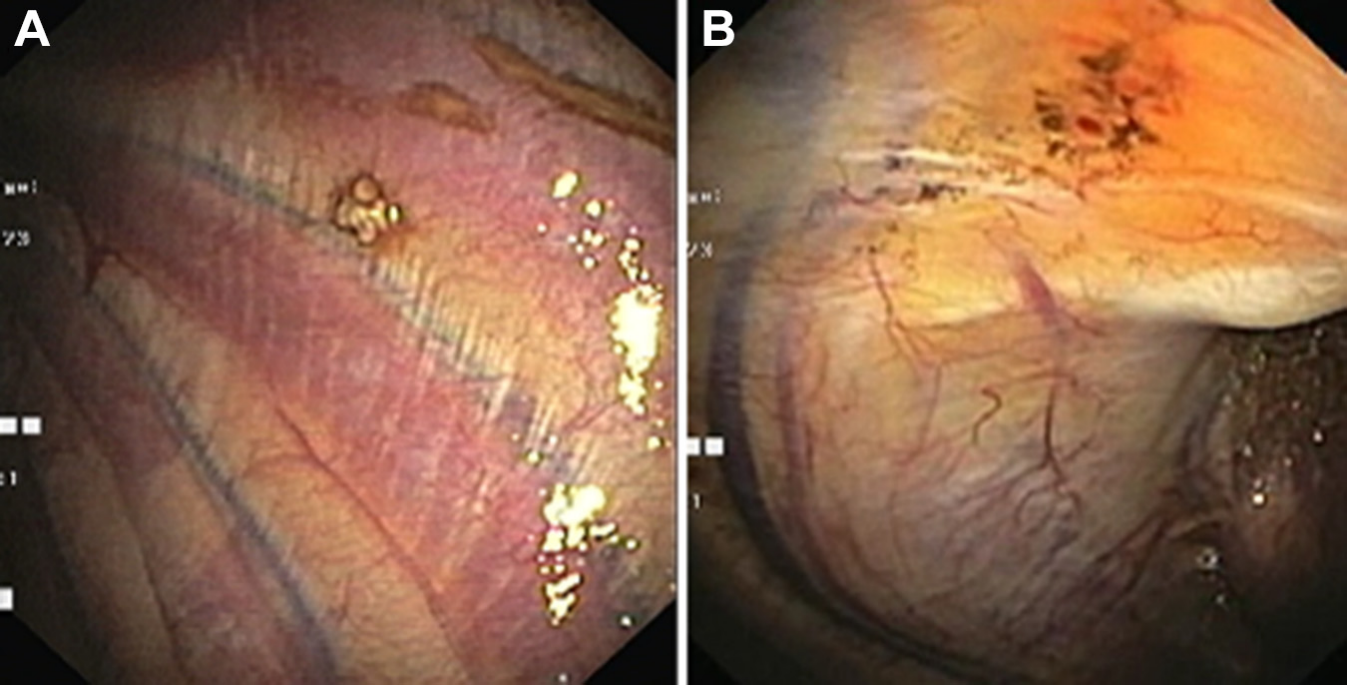
Medical thoracoscopy reveals yellow to dark brown nodules on the parietal pleural surface.

**Figure 4:**
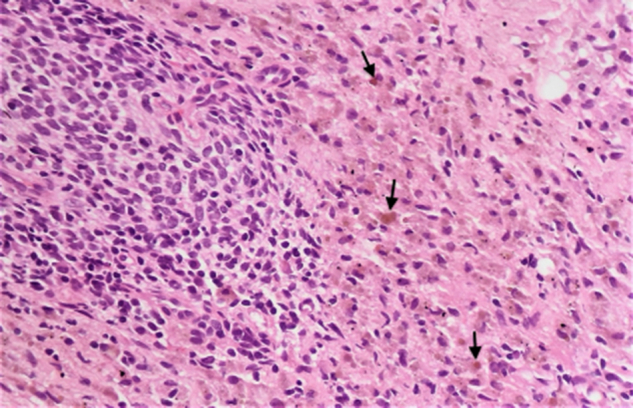
Microscopy shows the presence of islands of spindle cells (endometrial stromal cells) with collections of hemosiderin-laden macrophages (arrows) (hematoxylin-eosin stain, original magnification ×400).

**Figure 5:**
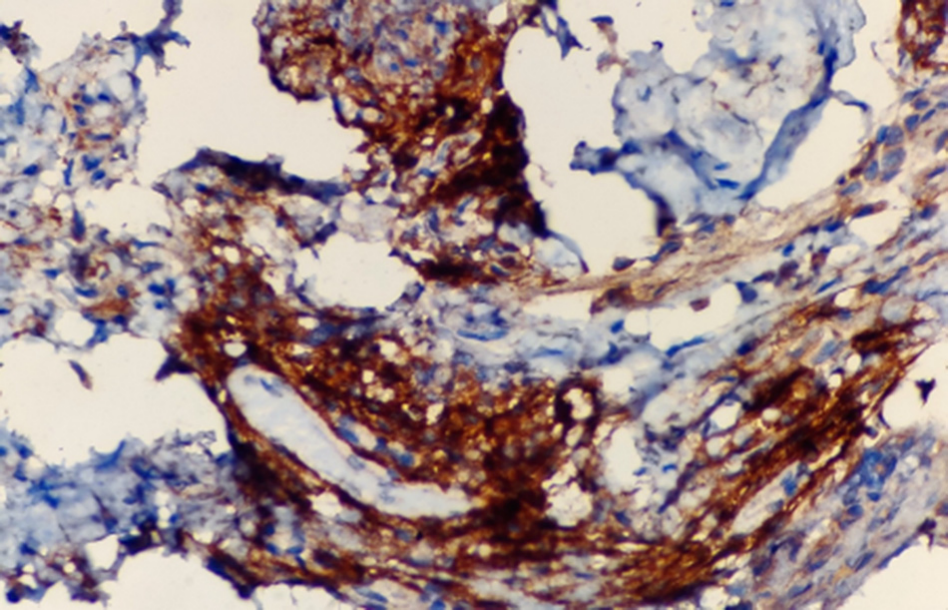
Stromal cells are positive for CD10 (immunohistochemistry, original magnification ×400).

**Figure 6:**
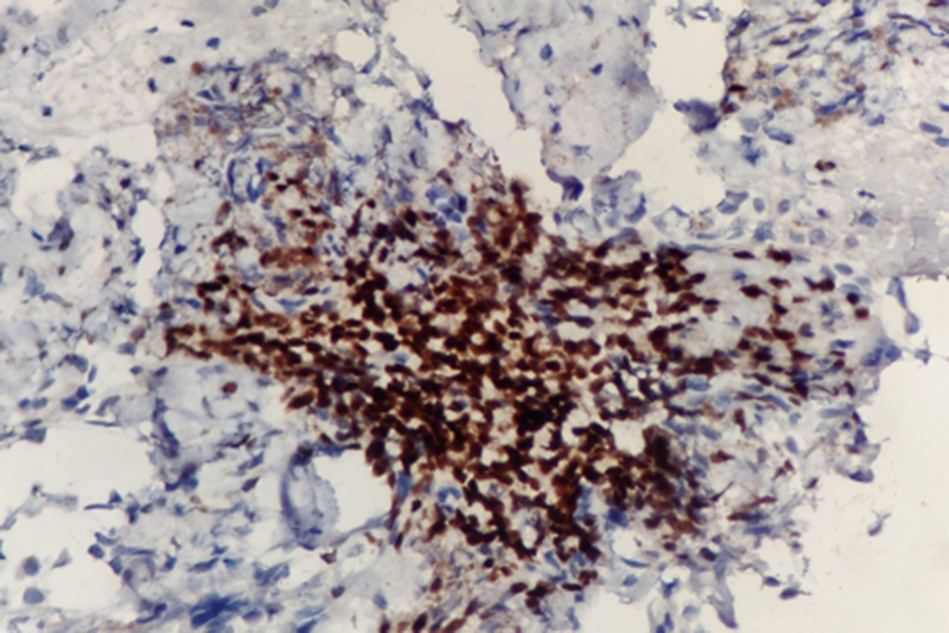
Stromal cells are positive for estrogen receptor (immunohistochemistry, original magnification ×400).

Initial laboratory investigation revealed a hemoglobin level of 10 g/dL, a WBC count of 10.8 × 10^9^/L (differential levels are as follows: neutrophils 56%, lymphocytes 36%, eosinophils 4%, monocytes 4%), and a platelet count of 661 × 10^9^/L. Blood metabolic panel, including renal and liver parameters, was normal. Chest radiograph revealed near-complete opacification of the right hemithorax with a contralateral shift of the heart, indicating a massive pleural effusion (Fig 1). A CT scan of the chest showed only pleural effusion without any pleural thickening or nodules (Fig 2). Transvaginal ultrasonography revealed a right ovarian cystic lesion of size 2.4 × 2 cm^2^, no findings on the left ovary, and a relatively well-defined hyperechoic lesion measuring 1.7 × 2.3 cm^2^ in the pouch of Douglas with minimal ascites in the pelvis. A MRI scan of the abdomen corroborated the ultrasound findings. Thoracentesis revealed hemorrhagic fluid, an exudate with a low adenosine deaminase level. Pleural fluid cytology was negative for malignant cells (Table 1). A flexible bronchoscopy excluded an endobronchial lesion. Given the undiagnosed pleural effusion, a medical thoracoscopy was performed, which revealed yellow to dark brown nodules measuring between 2 and 3 cm over the posterior parietal pleura and on the surface of the diaphragm (Fig 3). Thoracoscopic biopsy demonstrated hemosiderin-laden macrophages, tiny areas of endometrial stroma, and a focal collection of foamy histiocytes and foreign body giant cells (Fig 4). Immunohistochemistry showed positive staining for CD10 (Fig 5) and estrogen receptor (Fig 6).

**Table 1:** Pleural Fluid Characteristics of the Reported Case

Characteristic	Description
Color	Hemorrhagic
Type of fluid	Exudative Total protein: 4.3 g/dL Pleural fluid protein/serum protein ratio: 0.51 Lactate dehydrogenase: 490 units/L (serum lactate dehydrogenase was 418 units/L)
Cytology	Macrophage: 80% Lymphocyte: 15% Polymorph: 5%
Malignant cells/endometrial glands	Negative
Adenosine deaminase	20.42 units/L
Glucose	62 mg/dL

*What is the diagnosis?*

*Diagnosis:* Pleural endometriosis with endometriosis-related pleural effusion

## Discussion

Endometriosis is the presence of endometrium-like tissue outside the uterus, often associated with a chronic inflammatory response. The pelvis and peritoneal cavity are the most common sites for endometriosis. An estimated 5% to 10% of women in their childbearing age develop pelvic endometriosis. They usually present with pain in the abdomen, dysmenorrhea, abnormal uterine bleeding, and infertility.

Endometriosis outside the abdominopelvic cavity is rare. The most common site of extrapelvic endometriosis is the thoracic cavity, affecting the pleura, lungs, and diaphragm. Thoracic endometriosis most commonly presents as pneumothorax followed by hemothorax, hemoptysis, and lung nodules. Most often, although not always, these symptoms develop in association with menstruation and predominantly affect the right hemithorax. Concomitant pelvic endometriosis is commonly seen.

Endometriosis-related pleural effusion is a rare manifestation of thoracic endometriosis. Most patients are young (aged < 40 years), have concomitant pelvic endometriosis, and are nulliparous. The median duration between the onset of symptoms and diagnosis of pleural effusion is usually 6 months. The most common symptom is dyspnea, followed by chest pain and abdominal pain. The symptoms coincide with menstruation in most patients. The effusion is usually unilateral and right-sided in nearly 90% of patients. More than one-half of patients present with a large effusion. Concomitant pneumothorax can be seen in one-third of patients. The pleural fluid is exudative and appears bloody in almost all patients. The pleural fluid glucose level is usually > 60 mg/dL. Limited data suggest that the adenosine deaminase level can be low or high (> 35 units/L) in equal proportions of patients.

Various theories are proposed to explain the pathophysiology of endometrial deposits in the pleural space. According to Sampson’s theory of retrograde menstruation, endometrial cells travel backward through the fallopian tubes and into the peritoneal cavity. In the peritoneal cavity, there is a predictable flow of fluids and cells from the pelvis to the right subdiaphragmatic area through the right paracolic gutter while deviating away from the left hemidiaphragm due to obstruction of flow by the falciform and phrenicocolic ligaments. Afterward, the endometrial cells may get implanted in the diaphragm or migrate to the right pleural cavity through congenital defects or acquired fenestrations in the diaphragm. The preponderance of congenital defects in the right hemidiaphragm and the underlying liver exerting the piston effect may explain the preferential occurrence of endometriosis in the right hemithorax. The coelomic metaplasia theory postulates the origin of the endometrial tissue from the mesothelial cells lining the pleura, and estrogen may have a role in this metaplastic transformation. Furthermore, lymphatic and hematogenous dissemination of endometrial cells can lead to endometriosis in distant organs.

Diagnosis of endometriosis-related pleural effusion is challenging and often requires a high index of suspicion. Clinically, one may suspect endometriosis as the cause of a pleural effusion when the effusion is recurrent, right-sided, and associated with menstruation. However, definitive diagnosis requires demonstrating endometrial tissue in the fluid cytology or pleural biopsy. Pleural fluid cytology has a lower yield (9%) than pleural biopsy. The yield of pleural fluid cytology will likely improve if the pleural fluid is collected during menstruation and the cytopathologist is informed of the possibility of pleural endometriosis. A thoracoscopic biopsy is superior to a closed pleural biopsy in diagnosing pleural endometriosis. Typically, endometrial deposits in the pleura and diaphragm appear dark brown to violet, and size ranges from a few millimeters to 1 cm. Imaging modalities (eg, CT scan, MRI scan) can aid the diagnosis by excluding other diseases and delineating diaphragmatic endometrial deposits.

Histologically, the triad of endometrial glands, stroma, and hemosiderin-laden macrophages is considered diagnostic but seen infrequently in thoracic endometriosis. Furthermore, the diagnosis remains challenging when only small foci of endometrial stroma are found in lung or pleural tissue. Sometimes, it is difficult to distinguish endometrial stroma and inflammatory cells by histomorphology alone. Given these challenges, immunohistochemistry is crucial for the diagnosis of thoracic endometriosis. Generally, estrogen receptor, progestin receptor, and CD10 immunostaining are performed to confirm endometriosis. The estrogen and progestin receptors are expressed in large proportions of endometrial stroma and endometrial gland, whereas CD10 is a marker of endometrial stroma but not endometrial gland. Therefore, a positive estrogen receptor, progestin receptor, and CD10 are required for the identification of the endometrial stroma, whereas estrogen and progestin receptor positivity are required for the identification of the endometrial gland. CD10 has a sensitivity of nearly 96% in detecting endometrial stroma. Furthermore, two staining patterns are recognized for estrogen and progestin receptors—scattered and aggregated patterns. Notably, a scattered pattern staining is also identified in many cases of resected specimens of spontaneous pneumothorax in men. Therefore, merely identifying scattered patterns of staining for estrogen and progestin receptors in women is insufficient to diagnose pleural endometriosis. In an appropriate clinical context, a positive estrogen and progestin receptor in an aggregated pattern, along with positive CD10, is essential to confirm the diagnosis of pleural endometriosis.

Management of endometriosis-related pleural effusion includes treatment with antigonadotropic agents and surgery for the risk reduction of recurrence. Antigonadotropic agents act by decreasing endogenous estrogen production, leading to atrophy of endometrial tissue. Some of the agents that are used include cyclic or continuous oral contraceptives, dienogest, danazol, cyproterone acetate, and gonadotropin-releasing hormone agonists (eg, leuprolide). To our knowledge, there is no head-to-head comparison between various agents. Usually, the treatment is continued for 6 to 24 months. Recently, dienogest has shown promising results in managing endometriosis. When taken continuously, it inhibits systemic gonadotropin secretion and has local antiproliferative and antiinflammatory effects on endometriotic lesions. Furthermore, long-term treatment with dienogest 2 mg/d is associated with significant improvements in physical, mental, social, emotional, and general health parameters in women with endometriosis. The drug has been shown to be well tolerated and has been safely administered for up to 5 years in patients with endometriosis.

Medical therapy alone is associated with a higher recurrence rate. Therefore, surgical procedures are often performed upfront or on medical treatment failure. Video-assisted thoracic surgery remains the procedure of choice in managing thoracic endometriosis. It can detect blebs, bullae, and endometrial implants on the diaphragm, lungs, and visceral and parietal pleura. Furthermore, the surgeon can perform definitive surgery in the same setting, including pleurodesis. Hysterectomy and bilateral salpingo-oophorectomy may be performed in women who do not want to have pregnancy in future.

### Clinical Course

The lung remained unexpanded after a week of thoracoscopy. The chest tube was kept in situ, and negative suction was applied. Two weeks later, a complete expansion of the lung occurred, and the chest drain was removed. The patient has initiated oral dienogest 2 mg once daily and is scheduled for periodic assessment of the pleural and presumed pelvic endometriosis. She was doing well in her last follow-up 5 months after starting treatment.

## Clinical Pearls

1. *Endometriosis-related pleural effusion is a rare manifestation of thoracic endometriosis. It usually affects young women in their reproductive years. Concomitant pelvic endometriosis is seen in most patients and is an important clue to suspect pleural endometriosis.*
2. *Pleural effusion is usually exudative and hemorrhagic and mostly occurs on the right side. Pleural fluid cytology is usually noncontributory. The diagnosis is usually made by closed or thoracoscopic pleural biopsy.*
3. *Treatment includes progestins and gonadotropin-releasing hormone analogs that are usually given for 1 to 2 years.*
4. *Medical therapy is associated with a high recurrence rate. Video-assisted thoracoscopic surgery remains the modality of choice for surgical management of thoracic endometriosis.*

## Financial/Nonfinancial Disclosures

None declared.
